# Soluble Urokinase Receptor as a Promising Marker for Early Prediction of Outcome in COVID-19 Hospitalized Patients

**DOI:** 10.3390/jcm10214914

**Published:** 2021-10-24

**Authors:** Filomena Napolitano, Gaetano Di Spigna, Maria Vargas, Carmine Iacovazzo, Biagio Pinchera, Daniela Spalletti Cernia, Margherita Ricciardone, Bianca Covelli, Giuseppe Servillo, Ivan Gentile, Loredana Postiglione, Nunzia Montuori

**Affiliations:** 1 Department of Translational Medical Sciences and Center for Basic and Clinical Immunology Research (CISI), University of Naples Federico II, 80131 Naples, Italy; filomena-napolitano88@hotmail.it (F.N.); gaetanodispigna@inwind.it (G.D.S.); danielaspalletticern@libero.it (D.S.C.); mricciard@yahoo.it (M.R.); bianca.covelli@unina.it (B.C.); loredana.postiglione@unina.it (L.P.); 2 Department of Neurosciences, Reproductive and Odontostomatological Sciences, University of Naples Federico II, Via Pansini 5, 80131 Naples, Italy; vargas.maria82@gmail.com (M.V.); iacovazzo@tin.it (C.I.); giuseppe.servillo@unina.it (G.S.); 3 Department of Clinical Medicine and Surgery-Section of Infectious Diseases, University of Naples Federico II, Via Pansini 5, 80131 Naples, Italy; biapin89@virgilio.it (B.P.); ivan.gentile@unina.it (I.G.)

**Keywords:** biomarkers, COVID-19, suPAR, sepsis, ARDS, complement system, risk stratification in the emergency room

## Abstract

The Coronavirus disease 2019 (COVID-19), caused by SARS-CoV-2, has rapidly spread to become a global pandemic, putting a strain on health care systems. SARS-CoV-2 infection may be associated with mild symptoms or, in severe cases, lead patients to the intensive care unit (ICU) or death. The critically ill patients suffer from acute respiratory distress syndrome (ARDS), sepsis, thrombotic complications and multiple organ failure. For optimization of hospital resources, several molecular markers and algorithms have been evaluated in order to stratify COVID-19 patients, based on the risk of developing a mild, moderate, or severe disease. Here, we propose the soluble urokinase receptor (suPAR) as a serum biomarker of clinical severity and outcome in patients who are hospitalized with COVID-19. In patients with mild disease course, suPAR levels were increased as compared to healthy controls, but they were dramatically higher in severely ill patients. Since early identification of disease progression may facilitate the individual management of COVID-19 symptomatic patients and the time of admission to the ICU, we suggest paying more clinical attention on patients with high suPAR levels.

## 1. Introduction

The Coronavirus disease 2019 (COVID-19) is a viral infection caused by Severe Acute Respiratory Syndrome Coronavirus 2 (SARS-CoV-2) that has spread globally, resulting in the ongoing pandemic [1,2]. SARS-CoV-2 is transmitted mainly through airways; the median incubation period is 5.1 days before symptoms onset, but most infected individuals are asymptomatic or have mild illness [3]. When admitted to hospital, COVID-19 patients show a broad spectrum of clinical manifestations that include fever, dry cough, and fatigue, often with pulmonary involvement. Severe COVID-19 cases develop severe acute respiratory distress syndrome (ARDS), sepsis, and multiorgan dysfunction, and are admitted to the intensive care unit (ICU) [4].

The highest risk factors for severe COVID-19 illness are increased age, male sex and comorbidities such as diabetes, hypertension, chronic kidney failure and cardiac disease [5,6,7].

The pathogenesis of COVID-19 clinical manifestations is not completely clarified, but includes loss of control of proinflammatory cytokines production, vascular leakage and impaired T cell activation. Among the inflammatory mediators, TNF-α, IL-1β, IL-6, IL-8, IL-10, reactive oxygen species (ROS) and chemokines have shown to play a crucial role [8,9,10].

With ICU operating at maximum capacity during this time, it is crucial to identify patients with an increased risk of adverse outcomes and patients who can be safely discharged. Therefore, several routine biomarkers have been evaluated in COVID-19 patients. These include C-reactive protein (CRP), procalcitonin (PCT) and IL-6, but, overall, their measurement does not always provide indications with high certainly of evidence in predicting disease progression or mortality [11].

The urokinase receptor (uPAR) is a component of the fibrinolytic system acting as a cofactor for plasminogen activation through its ligand urokinase-type plasminogen activator (uPA) [12].

uPAR is structurally a glycosylphosphatidylinositol (GPI) glycoprotein anchored to the surface of several cell lines, including immune cells, especially neutrophils, monocytes, and macrophages. It consists of three homologous domains (DI, DII and DIII), each of approximately 90 amino acids [13].

It has long been believed that the role of uPAR was only to concentrate the uPA activity on the cell surface. To date, a plethora of physiological and pathological functions has been attributed to this receptor in many events that require extracellular matrix (ECM) remodeling [14]. To perform its multiple functions, uPAR contacts different molecular partners on the cell surface, creating a broad interaction network, also named “uPAR interactome”. The list of its ligands includes proteins that are structurally and functionally different such as vitronectin (VN), and membrane-bound partners as integrins and growth factor receptors, through which uPAR stimulates cell adhesion, migration and proliferation [15,16,17,18,19,20]. *N*-formyl peptide receptors (FPRs), belonging to the GCRP superfamily, were also shown to interact with uPAR. This interaction occurs after a proteolytic cleavage of DI by different enzymes or a conformational change by uPA binding, thus determining the exposure of the SRSRY motif located in the linker region between DI and DII. The cleaved uPAR (DII-DIII uPAR) is unable to bind uPA and VN, but is still able to interact with FPRs, thereby promoting cell migration and chemotaxis [15,21,22].

After the removal of the GPI anchor by proteases or phospholipases, both full-length and cleaved uPAR shed from the cell membrane and exist as a soluble form (suPAR) that is detectable in human body fluids [23]. More recently, suPAR has attracted scientific interest because it seems to discriminate better than some other biomarkers such as C-reactive protein (CRP) and procalcitonin (PCT) among patients with different severities of illness. In a wide population of acute medical patients, suPAR is strongly associated with disease severity and mortality, suggesting that suPAR could be used in the clinic as a prognostic indicator [24]. Several studies have indicated that serum suPAR levels correlate with the severity of infection and have reported a strong association with mortality in patients with malaria, tuberculosis, and human immunodeficiency virus (HIV) infection [25,26,27].

Therefore, we analyzed serum suPAR levels in COVID-19 patients with different disease severity to support the clinical value of this biomarker as a prognostic index.

The complement system is well known for promoting immune cell activation and proinflammatory state [28,29]. Anaphylatoxins C3a and C5a are able to activate neutrophils, mast cells, monocytes/macrophages, basophils, eosinophils, T and B lymphocytes [30]. Recent reports refer to patients with severe COVID-19 showed prominent complement activation, and some of these individuals have been treated with complement inhibitors [31,32,33]. However, it is still not completely clear whether complement serves as a friend or foe in the COVID-19 [34].

Since we hypothesized a pathogenetic interplay between complement system and the uPA/uPAR system [35], another purpose of our work is to analyze serum levels of complement proteins in COVID-19 patients.

## 2. Materials and Methods

### 2.1. Study Participants and Study Design

A single-center cohort study was conducted at the University of Naples Federico II in the periods of the first wave (from March until June 2020) and the second wave (from September until December 2020) of COVID-19 pandemic. The inclusion criteria included: (1) adult patients (≥18 years old); (2) laboratory determination of SARS-CoV-2 infection defined a positive result of RT-PCR assay on nasal and pharyngeal swabs; and (3) the blood samples collected within 48 h of admission and stored for the molecular biomarkers analyses. Ten control subjects (mean age 55 ± 12 years; M:F 2:8) with negative swabs and without a family history of COVID-19 were recruited from employees of University of Naples Federico II.

Eighteen cases of SARS-CoV-2 infection admitted to the Infectious Diseases Unit at the University of Naples Federico II and 52 subjects admitted in the Intensive Care Unit (ICU) of the University of Naples Federico II during the second wave of COVID-19 pandemic were enrolled in a prospective manner. ICU patients were classified according to the exitus into two groups, survivor and non-survivor, numerically comparable.

In order to confirm the prognostic value of suPAR, a retrospective analysis on stored samples from the first pandemic wave recorded in Italy was performed. To this aim, 18 patients from the Infectious Diseases Unit and 16 patients from the ICU, hospitalized at the University of Naples Federico II, were enrolled in the study.

Clinical presentation, laboratory test results, and disease outcomes were collected from each patient recruited in the study. The staff of Clinical Pathology Laboratory, at the Department of Translational Medical Sciences, University of Naples Federico II, was unaware of clinical patients’ outcome and was therefore blinded.

### 2.2. SARS-CoV-2 Testing

The determination of SARS-CoV-2 infection was performed on all patients in routine diagnostics in the Clinical Pathology Laboratory at the Department of Translational Medical Sciences, University of Naples Federico II. The nasopharyngeal swabs were analyzed by using Abbott Real-Time SARS-CoV-2 kit as described in the instructions of the manufacturer, on the Alinity platform. The results were considered as positive when the Ct value on N and/or on ORF1b genes was equal or less than 37 [36].

### 2.3. Measurements Methods

suPAR was measured using the BioVendor Human suPAR ELISA on the automated analyzer Triturus System (GRIFOLS, Vicopisano, Italy). The intra- and inter-assay coefficients of variation were ˂5%. IL-6 determination was performed by Chemiluminescent Immunoassay (CLIA) by Immulite 2000 (Siemens Healthcare Diagnostics, Milan, Italy). The analysis of C3, C4 and C1inh was performed through nephelometric detection by BN ProSpec System (Siemens Healthcare Diagnostics, Italy). The intra- and inter-assay coefficients of variation were ˂5%.

C-reactive protein (CRP) was determined on serum samples using an immunoturbidimetric method by Architect ci8200 (Abbott Laboratories, Illinois, USA). The reference range for CRP has been measured within the 0.5 to 1 mg/dL.

Procalcitonin was detected using chemiluminescence immunoassay on the bioMérieux VIDAS analyzer. The reference value of procalcitonin in adults is 0.15 ng/mL or less.

### 2.4. Statistical Analysis

The data were presented as mean and standard deviation (SD). Categorical variables were presented as number (*n*) and percentages (%). Normally distributed continuous variables were compared with Student t-test and the Fisher’s exact test to compare dichotomic variables. *p*  <  0.05 was considered statistically significant.

In order to create a prognostic rule, receiver operating curve (ROC) analysis was done with suPAR, IL-6 and CRP on day 1 of hospitalization as independent variables to predict unfavorable outcome. Analysis of ROC curves was performed with the MedCalc software Version 11.5.1 (Medcalc Software Ltd., 8400 Ostend, Belgium). Statistical significance of the area under the ROC curves (AUC) was calculated against the null hypothesis AUC = 0.5 as recommended by DeLong et al. [37]. Threshold values were determined by the farthest point from the bisector of the ROC curve.

## 3. Results

### 3.1. Experimental Design and Patients’ Enrollment

**Figure 1:**
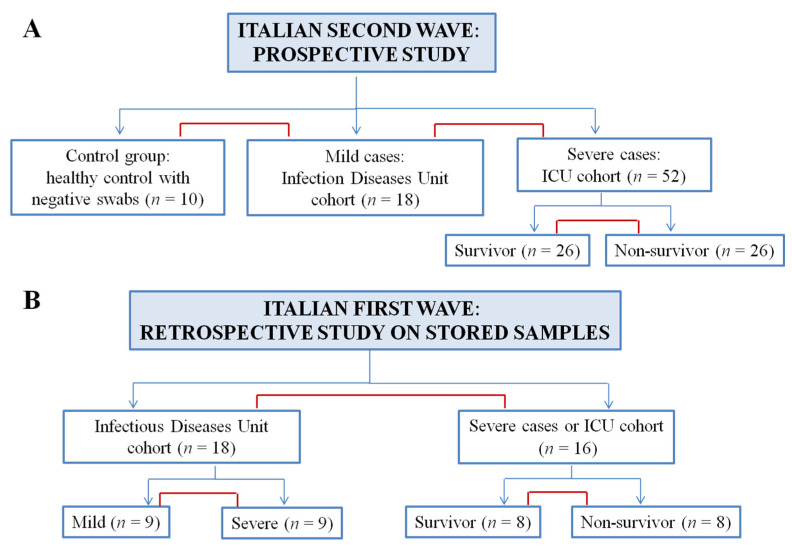
Flow diagram for patients’ enrollment and study design. (**A**) In the first phase of this study ten control subjects, eighteen patients from Infectious Diseases Unit and fifty-two ICU patients were enrolled in a prospective manner. Control vs. mild cases, mild cases vs. ICU patients, survivor vs. non-survivor were compared. (**B**) Retrospective analysis of patients hospitalized during the first wave included eighteen patients from Infectious Diseases Unit stratified in nine mild patients and nine severe patients, and sixteen ICU patients stratified in eight survivors and eight non-survivors. First comparison was performed between mild and severe patients. Thus, Infectious Diseases Unit vs. ICU cohorts and survivor vs. non-survivor were compared.

Patients admitted to our hospital during the Italian second wave of the COVID-19 pandemic were enrolled in a prospective manner. In these series of experiences, ten controls with negative swabs, eighteen patients admitted to the Infectious Diseases Unit with mild illness, and fifty-two severe cases were enrolled and compared as described in Figure 1A.

In the second phase of this study, we performed a retrospective analysis on patients hospitalized during the first wave, using stored samples available in our laboratories. As shown in Figure 1B, eighteen patients were selected from the Infectious Diseases Unit using random sampling stratified by mild and severe trajectories. Sixteen patients were selected from the ICU using random sampling stratified by exitus (survivors, non-survivors). First comparison involved the two cohorts of Infectious Diseases Unit and ICU patients; then mild vs. severe (Infectious Diseases Unit) and survivor vs. non-survivor group were compared. This sampling approach was used to ensure adequate representation for all COVID-19 patients admitted to our hospital during the pandemic’s first wave.

### 3.2. suPAR Serum Levels in Mild Cases of COVID-19 during Italian Second Wave

Since 1 September 2020, 18 adult patients admitted to the Infectious Diseases Unit at the University of Naples Federico II, with acquired pneumonia and molecular documentation of SARS-CoV-2 in respiratory secretions, were enrolled in the study. The main clinical features are reported in Table 1. This patient cohort had a mean age of 59 ± 16 and consisted of 77% male. The disease severity was determined by the NIAD ACTT-1 (National Institute of Allergy and Infectious Diseases Adaptive COVID-19 Treatment Trial-1) Clinical Status Ordinal Scale [38]. Based on this score, we classified each patient with the infection into one of eight categories: (1) not hospitalized, no limitations on activities; (2) not hospitalized, limitation on activities, and/or requiring home oxygen; (3) hospitalized, not requiring supplemental oxygen and no longer requires ongoing medical care (if hospitalization extended for infection-control purposes); (4) hospitalized, not requiring supplemental oxygen; requiring ongoing medical care (COVID-19 related or otherwise); (5) hospitalized, requiring supplemental oxygen; (6) hospitalized, requiring noninvasive ventilation or high-flow oxygen devices; (7) hospitalized, requiring invasive mechanical ventilation or ECMO; and (8) death. The patients analyzed in this first experience presented a score between 3 and 4, indicating a mild disease trajectory as supplemental oxygen has only been required during hospitalization. Among patients, none had been transferred to an intensive setting or had died.

From a clinical laboratory perspective, the analysis of inflammatory markers in Table 1, including C-reactive protein (CRP), procalcitonin and IL-6, confirmed the need of a new decision marker for early prediction of disease trajectory. In fact, the patient cohort exhibited normal levels of CRP and procalcitonin, while the IL-6 levels were dramatically higher and did not reflect the mild disease trajectory.

**Figure 2:**
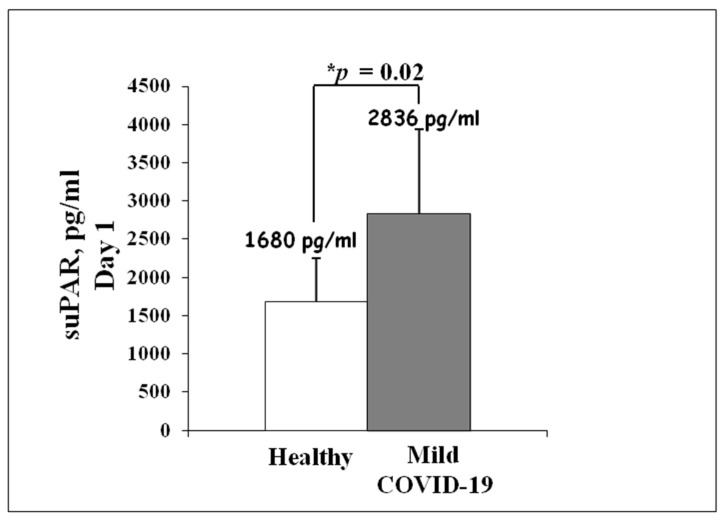
Analysis of serum suPAR levels in mild cases of COVID-19 during Italian second wave. suPAR measurements were performed by ELISA method on serum samples within 24 h of admission to Infectious Disease Unit (grey column). Ten healthy subjects were enrolled as a control (white column). Asterisk indicates statistical significance between healthy control and COVID-19 patients, determined by the Student *t*-test (* *p* < 0.05).

In order to analyze suPAR levels in COVID-19 patients with a mild disease trajectory, measurements were performed by ELISA method on serum samples within 24 h of admission. Ten healthy subjects were enrolled as a control. As shown in Figure 2, mildly ill patients had significantly higher serum suPAR concentrations at admission (2836 ± 1102 pg/mL) than healthy control (1680 ± 567 pg/mL). These preliminary data encouraged the hypothesis that the study of suPAR might provide an efficient biomarker of disease progression.

### 3.3. Presenting Characteristics and Analysis of suPAR Serum Levels in Severe Cases of COVID-19 during Italian Second Wave

In the same period (second wave), 52 adult patients hospitalized in the Intensive Care Unit (ICU) of the University of Naples Federico II were enrolled in the study, according to the outcome: survivors (*n* = 26) and non-survivors (*n* = 26). The main clinical features are shown in Table 2. Overall, the cohort had a mean age of 70 ± 11 years and consisted of 82% male. The computing of the Charlson comorbidity index, which provides an overall measure of an individual patient’s complexity [39], showed that the score was significantly higher in the non-survivors (5.3 ± 2.9) than in the survivor group (3.3 ± 3). Hypertension (63%) was the most common comorbidity, followed by diabetes (21%) and chronic kidney failure (21%).

Overall, high levels of inflammatory biomarkers, including CRP, PCT and IL-6, were found in the entire ICU cohort, but there was no statistically significant difference between survivor and non-survivor groups. Moreover, it was interesting to observe that IL-6 levels in the ICU cohort (65.9 ± 92 pg/mL; Table 2) did not differ from IL-6 levels estimated in the Infectious Diseases Unit cohort (63.7 ± 84 pg/mL; Table 1) showing mild clinical symptoms.

**Figure 3:**
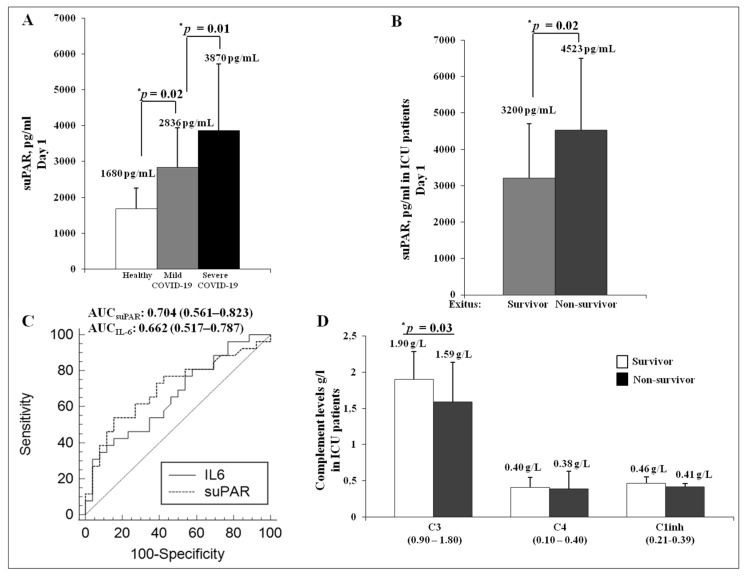
Analysis of serum suPAR levels in severe cases of COVID-19 during the Italian second wave. (**A**) serum suPAR levels in healthy controls (white column), mild cases (grey column) and severe cases of COVID-19 (black column); * *p* < 0.05. (**B**) analysis of serum suPAR levels in severe cases of COVID-19 hospitalized in the Intensive Care Unit (ICU) and classified in survivors (light grey column) and non-survivors (dark grey column). Asterisk indicates statistical significance between survivors and non-survivors, determined by the Student *t*-test (* *p* < 0.05). (**C**) Receiver Operating Curve (ROC) analysis of IL-6 and suPAR to define unfavorable outcome in the ICU cohort. Areas under the curve (AUCs) and 95% confidence intervals are shown. (**D**) analysis of the complement system in the ICU cohort patients classified in survivors (white column) and non-survivors (black column). C3, C4 and C1 inhibitor (C1inh) measurement was performed through nephelometric technique. Asterisk indicates statistical significance between survivors and non-survivors, determined by the Student *t*-test (* *p* < 0.05).

Serum suPAR levels were analyzed in a cohort of severe cases of COVID-19 at first day of admission in ICU. As shown in Figure 3A, the total cohort (*n* = 52) showed a dramatically significant increase of suPAR levels (3870 ± 1854 pg/mL) as compared to healthy controls (1680 ± 567 pg/mL) and to patients with a mild form of COVID-19 (2836 ± 1102 pg/mL). The data suggested that suPAR strongly correlated with the severity of the disease. Indeed, the non-survivor group exhibited higher levels of serum suPAR (4523 ± 1976 pg/mL) than the survivor group (3200 ± 1488 pg/mL), suggesting that suPAR may be predictive of exitus (Figure 3B).

Moreover, the receiver operator curve (ROC) analysis indicated that the areas under the curve (AUCs) are much greater for serum suPAR (0.704; *p* < 0.006) than for IL-6 (0.662; *p* < 0.03). Coordinate points of ROC for suPAR analysis defined 4230 pg/mL as a cutoff and a specificity of greater than 84.6% to predict exitus (Figure 3C).

### 3.4. Analysis of the Complement System in the ICU Cohort

The uPA/uPAR system is involved in the complement activation by promoting plasminogen conversion to plasmin that is able to activate the classic complement system or to act directly on C3 and C5, thus producing, respectively, C3a and C5a. This new knowledge clarifies some fundamental molecular aspects of the link between the fibrinolytic system and the complement system in the Angioedema pathogenesis [35].

Hence, we analyzed the serum levels of complement proteins in order to verify a possible connection between the fibrinolytic system and the complement system in the COVID-19 pathogenesis. To this end, we decided to measure C3, C4 and C1 inhibitor (C1inh) in ICU patients. Interestingly, no significant difference was found in C3, C4 and C1inh levels among ICU patients and healthy control (data not shown). However, among ICU patients, the non-survivors showed a significant reduction in C3 levels as compared to the survivor group, whereas this difference was not observed for C4 and C1inh levels (Figure 3D).

Based on this data, the ICU cohort did not exhibit functional defects in the upstream of the complement cascade (C4 analysis) or in the regulation of this system (C1inh analysis). The C3 reduction in non-survivors could be associated with suPAR higher levels; in fact, enhanced suPAR release may concur to reduce plasmin generation through competitive inhibition of uPA binding to the cell surface, thereby promoting the development of a hypofibrinolytic condition and hypercoagulable state [40].

On the other hands, it is plausible that the C3 levels could be consumed by high levels of suPAR in non-survivors serum, because suPAR itself, through uPA binding, could directly convert plasminogen into plasmin, thus promoting the continuous release of anaphylotoxins from C3 and C5. This hypothesis might explain the effectiveness of the pharmacological approach with a terminal complement inhibitors such as the antibodies Eculizumab or Ravulizumab [41,42].

### 3.5. Retrospective Analysis of suPAR Serum Levels in Hospitalized COVID-19 Patients during Italian First Wave

Aiming to confirm our results on suPAR prognostic significance, we studied stored samples from patients with mild/moderate COVID-19 (*n* = 18) admitted to the Infectious Diseases Unit during the period from March until June 2020. The main clinical characteristics and laboratory data of patients enrolled in this experience are shown in Table 3. The mean age of patients was 69 ± 16, and 88% were male. At first presentation, the evaluation of Clinical Status Ordinal Score indicated that severe COVID-19 was much more abundant in patients of the first wave than patients of second wave previously analyzed. The cohort exhibited a mean score of 5, indicating the need of nasal high-flow oxygen therapy or non-invasive mechanical ventilation for these patients. During hospitalization, three patients died while six patients were transferred to ICU. Laboratory data showed a dramatic increase of inflammatory markers compared to the normal range; in particular, this cohort presented elevated concentrations of IL-6 (254 ± 690 pg/mL).

**Figure 4:**
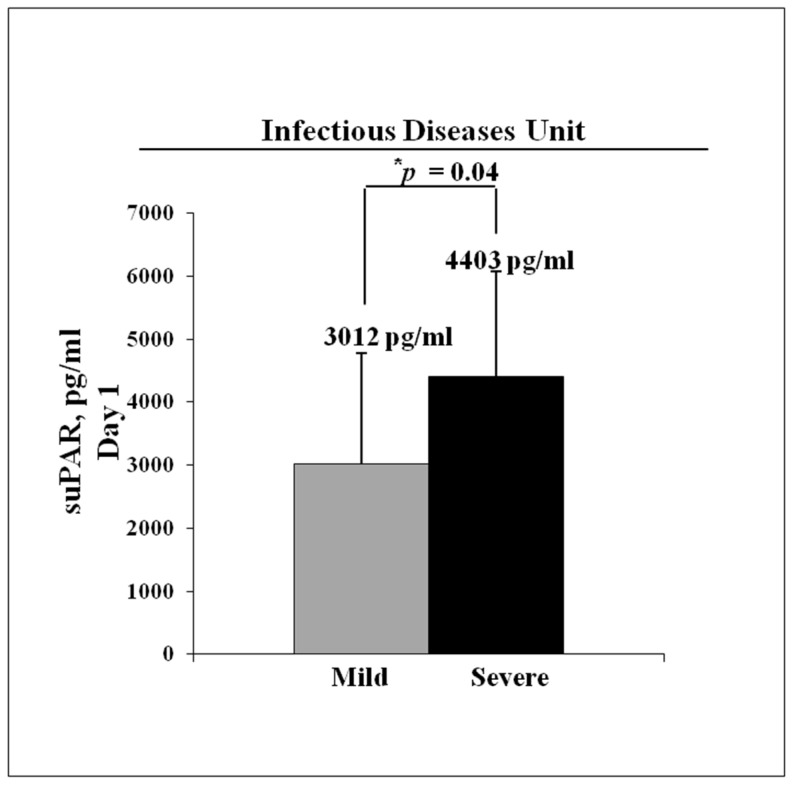
Analysis of serum suPAR levels in hospitalized COVID-19 patients during Italian first wave. suPAR measurements were performed by ELISA method on serum suPAR within 24 h of admission to Infectious Disease Unit. Asterisk indicates statistical significance between nine mild cases (grey column) and nine severe cases (black column) of COVID-19, determined by the Student *t*-test (* *p* < 0.05).

Overall, the cohort exhibited high suPAR levels (3671 ± 1817 pg/mL), as reported in Table 3, and the analysis of disease trajectory confirmed our hypothesis that suPAR correlated with COVID-19 severity. In fact, this cohort was partitioned into two groups showing mild disease trajectory (mild) or severe clinical course (severe). Mild patients occasionally required supplemental oxygen and were successfully discharged from the Infectious Diseases Unit. Instead, the patients with severe clinical disease were transferred from the Infectious Diseases Unit to the ICU to be subjected to the non-invasive or invasive mechanical ventilation. In this group, death in hospital occurred in three patients. When we performed the comparisons of inflammatory markers between mild and severe groups, we surprisingly found that CRP was significantly higher in mild patients than severe patients. The only biomarker among those analyzed capable at the first presentation of predicting the disease trajectory was suPAR. As shown in Figure 4, severe group presented a significant increase of suPAR levels (4403 ± 1665 pg/mL) as compared to mild patients (3012 ± 1766 pg/mL).

### 3.6. Retrospective Analysis of suPAR Serum Levels in Severe Cases of COVID-19 during Italian First Wave

To complete the analysis of suPAR expression in COVID-19 patients during the first wave of pandemic, we included in this study 16 critically ill patients hospitalized in the ICU and, classified in two numerically comparable groups, survivor and non-survivor. 

Overall, the mean age of the ICU cohort was 65 ± 13 and 82% (*n* = 13) was male. Three patients had diabetes, and eight patients were affected by hypertension. Chronic heart failure and chronic kidney disease affected, respectively, six and two patients of the ICU cohort (Table 4). Table 4 also shows the clinical differences between survivors and non-survivors of the cohort. The analysis of inflammatory markers showed that CRP could represent a useful prognostic marker as the cohort presented dramatic levels of CRP (192 ± 102 mg/mL) and could be predictive of mortality at first presentation in the ICU as non-survivors exhibited higher serum CRP levels (246 ± 116 mg/mL) than survivors (138 ± 48 mg/mL). IL-6 did not show any significant difference in levels between survivors and non-survivors in this cohort.

**Figure 5:**
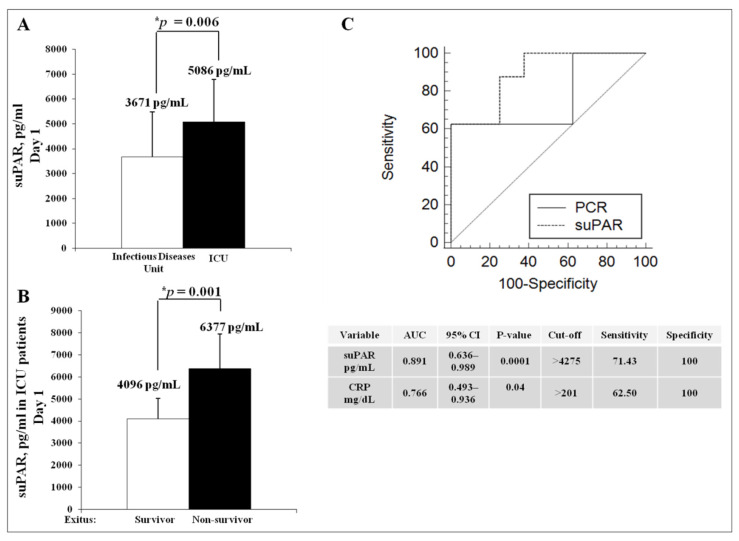
Analysis of serum suPAR levels in severe cases of COVID-19 during the Italian first wave. (**A**) serum suPAR levels in patients hospitalized in Infectious Disease Unit (white column) and ICU (black column). Significant difference between the means of two groups was determined by the Student *t*-test (* *p* < 0.05). (**B**) analysis of serum suPAR levels in severe cases of COVID-19 hospitalized in the Intensive Care Unit (ICU) classified in survivors (white column) and non-survivors (black column). Asterisk indicates statistical significance between two groups, determined by the Student *t*-test (* *p* < 0.05). (**C**) Receiver Operating Curve (ROC) analysis of CRP and suPAR to define unfavorable outcome in the ICU cohort. Areas under the curve (AUCs), 95% confidence intervals, cut-off, sensitivity and specificity are shown in the table.

As shown in Figure 5A, ICU patients exhibited a significant increase in suPAR concentrations (5086 ± 1709 pg/mL) in respect to patients admitted to the Infectious Diseases Unit (3671 ± 1871 pg/mL). Importantly, serum suPAR was significantly higher in non-survivors (6377 ± 1555) as compared to survivors (4096 ± 935), as shown in Figure 5B.

ROC curve was used to evaluate the potential predictive value of serum suPAR on prognosis at first presentation in-hospital. ROC analysis was done with suPAR and CRP of day 1 of hospitalization as independent variables to predict unfavorable outcomes. The ROC curves are shown in Figure 5C. The AUC of suPAR and CRP were 0.891 (95% CI 0.636–0.989) and 0.766 (0.493–0.936), with cut-off values of 4275 pg/mL and 201 mg/mL, respectively. Other exact values of the ROC curve analysis, including sensitivity and specificity, are shown in Figure 5C. Altogether, this analysis indicated that suPAR is a better predictor of death in individuals with severe COVID-19 in our study, much more than CRP.

## 4. Discussion

The early identification of COVID-19 individuals at low or high risk of serious illness may improve the patient stratification according to risk of mortality and severe disease development. However, currently admission blood biomarkers have only moderate predictive value for COVID-19 outcome and decision making in the clinical setting.

Here, we investigated the newly introduced inflammatory marker suPAR in hospitalized patients affected by different forms of COVID-19, from mild to severe disease or death.

Many authors have suggested uPA/uPAR system potential role as a main orchestrator of fatal progression of COVID-19, thereby inviting clinical laboratorists to analyze the suPAR levels in COVID-19 patients [43]. Indeed, several cohort studies in acutely presenting patients showed that suPAR has the best performance in predicting the need for non-invasive ventilation (NIV) or ICU admission [44,45]. Moreover, Azam TU et al. reported that the admission suPAR levels in patients hospitalized for COVID-19 are predictive of acute kidneys injury (AKI) and the need for dialysis [46].

The primary study endpoint was to define whether suPAR could represent a non-specific biomarker with predictive value for unfavorable outcomes in COVID-19 patients. Firstly, we observed an association between suPAR levels and disease presence in non-critical patients hospitalized in the Infectious Disease Unit during second wave of COVID-19 pandemic. Secondly, we found that suPAR was strongly associated with disease severity and mortality, as shown from the data obtained analyzing severe COVID-19 patients in respect to those affected by milder disease forms.

We find very attractive the possibility to use suPAR as an indicator for risk definition and patient stratification. Thus, we studied stored samples of 18 non-critical patients hospitalized in the Infectious Disease Unit and 16 critical patients hospitalized in the ICU during the first wave of pandemic. In non-critical patients hospitalized in the Infectious Disease Unit, elevated serum suPAR levels at first presentation were associated with the need of ICU admission and mechanical ventilation or death. Moreover, serum suPAR levels represented a promising biomarker of mortality in ICU patients.

As shown by ROC curve analysis, suPAR had the best prognostic performance respect to both IL-6 and CRP in the analyzed cohorts of infected patients. In our study, suPAR was compared to IL-6 levels in the second wave; indeed CRP was increased, but not significant difference among survivors and non-survivors in the ICU cohort was found. In the first wave, suPAR was compared to CRP because its levels were significantly different among survivors and non-survivors in the ICU cohort.

This study highlights several differences between the two waves of the pandemic. Laboratory data showed a lower inflammatory component, as demonstrated by IL-6, procalcitonin and PCR levels in the second wave. Significant differences in serum suPAR levels determined at hospital admission were closely associated with the subsequent disease severity in both COVID-19 waves, but in the first wave, this biomarker was much more augmented. It is conceivable that a higher use of corticosteroids during the second wave changed the disease trajectories.

Moreover, our study shows that serum suPAR may represent a promising biomarker of clinical severity, hospital and ICU admission, complications, mortality and could be useful in COVID-19 patient triage.

We acknowledge that there are several limitations of this study. First, whether suPAR is a useful therapeutic option will require further studies on a larger sample size. Second, baseline comorbidities affecting the enrolled patients might contribute to suPAR level increase and their impact could be analyzed by multivariable analysis in a larger number of patients. Third, further studies are required to assess an optimal cut-off for predicting outcomes and determining the need for intensive care.

## Figures and Tables

**Table 1:** Clinical characteristics and laboratory data of the Cohort including eighteen COVID-19 patients hospitalized in the Infectious Diseases Unit during Italian second wave and compared to ten healthy controls.

	Cohort (*n* = 18)	Healthy (*n* = 10)	*p*-Value *
**Clinical characteristics:**			
Age, average (SD)	59 (16)	55 (12)	0.228
Male, *n* (%)	14 (77)	2 (10)	0.005 *
Clinical Status Ordinal Scale	3.8 (1.15)		
Diabetes, *n* (%)	2 (11)		
Chronic heart failure, *n* (%)	1 (5.5)		
Chronic kidney disease, *n* (%)	-		
Died	0		
**Laboratory findings, average (SD):**			
CRP, mg/dL	6.75 (6.4)		
Procalcitonin, ng/mL	0.15 (0.15)		
IL-6, pg/mL	63.7 (84)	3.0 (0.80)	0.003 *
suPAR, pg/mL	2836 (1102)	1680 (567)	0.02 *

*p*-value for the comparison between the Cohort and Healthy controls. Asterisks (*) indicate statistical significance (*p* < 0.05). CRP, C-reactive protein. CRP, procalcitonin and IL-6 were measured at first presentation. CRP and procalcitonin data were unavailable for healthy controls.

**Table 2:** Clinical characteristics and laboratory data of ICU patients during the Italian second wave.

	ICU Cohort (*n* = 52)	Survivors (*n* = 26)	Non-Survivors (*n* = 26)	*p*-Value *
**Clinical characteristics**:				
Age, average (SD)	70 (11)	64 (12)	72 (9.8)	0.008 *
Male, *n* (%)	43 (82)	20 (76)	23 (88)	0.465
Charlson’s comorbidity index (DS)	4.3 (3.1)	3.3 (3)	5.3 (2.9)	0.01 *
Diabetes, *n* (%)	11 (21)	5 (19)	6 (23)	1.000
Hypertension, *n* (%)	33 (63)	17 (65)	16 (61)	1.000
Chronic heart failure, *n* (%)	1 (1.9)	-	1 (3.8)	1.000
Chronic kidney disease, *n* (%)	11 (21)	2 (7.6)	9 (34)	0.008 *
**Laboratory findings, average (SD):**				
CRP, mg/dL	119 (92)	99.9 (88)	139 (93)	0.06
Procalcitonin, ng/mL	10.2 (41)	6.7 (27.3)	13.7 (51.6)	0.271
IL-6, pg/mL	65.9 (92)	41.6 (44)	73.2 (120)	0.02 *
suPAR pg/mL	3870 (567)	3200 (1488)	4523 (1976)	0.004 *

ICU, intensive care unit; CRP, C-reactive protein. *p*-value for the comparison between survivor and non-survivor groups. Asterisks (*) indicate statistical significance (*p* < 0.05). CRP, procalcitonin and IL-6 were measured at first presentation.

**Table 3:** Clinical characteristics and laboratory data of hospitalized COVID-19 patients during the Italian first wave.

	Cohort (*n* = 18)	Mild (*n* = 9)	Severe (*n* = 9)	*p*-Value *
**Clinical characteristics:**				
Age, average (SD)	69 (16)	65 (17)	73 (15)	0.149
Male, *n* (%)	16 (88)	8 (89)	8 (89)	1.000
Clinical Status Ordinal Scale	5 (1.10)	4.6 (0.7)	5.5 (1.1)	0.03 *
Diabetes, *n* (%)	7 (39)	4 (44)	3 (33)	1.000
Chronic heart failure, *n* (%)	3 (17)	2 (22)	1 (11)	1.000
Died, *n* (%)	3 (11)	-	3 (33)	0.165
**Laboratory findings, average (SD):**				
CRP, mg/dL	8.5 (7.5)	12.42 (8.8)	3.4 (1.7)	0.004 *
Procalcitonin, ng/mL	0.25 (0.52)	0.29 (0.65)	0.10 (0.13)	0.196
IL-6, pg/mL	254 (690)	138 (341)	371 (931)	0.245
suPAR, pg/mL	3671 (1817)	3012 (1766)	4403 (1665)	0.04 *

*p*-value for the comparison between mild cases and severe cases of COVID-19 patients. Asterisks (*) indicate statistical significance (*p* < 0.05). CRP, procalcitonin and IL-6 were measured at first presentation.

**Table 4:** Clinical characteristics and laboratory data of ICU patients during the Italian first wave.

	ICU Cohort (*n* = 16)	Survivors (*n* = 8)	Non-Survivors (*n* = 8)	*p*-Value *
**Clinical characteristics**:				
Age, average (SD)	65 (13)	61 (12)	69 (14)	0.121
Male, *n* (%)	13 (82)	6 (75)	7 (87.5)	1.000
Diabetes, *n* (%)	3 (18.7)	1 (12.5)	2 (25)	1.000
Hypertension, *n* (%)	8 (50)	6 (75)	2 (25)	0.066
Chronic heart failure, *n* (%)	6 (37.5)	3 (37.5)	3 (37.5)	1.000
Chronic kidney disease, *n* (%)	2 (12.5)	-	2 (25)	0.233
**Laboratory findings, average (SD):**				
CRP, mg/dL	192 (102)	138 (48)	246 (116)	0.01 *
IL-6, pg/mL	85.25 (53)	96.37 (66)	74.13 (37)	0.184
suPAR, pg/mL	5086 (1709)	4096 (935)	6377 (1555)	0.001 *

*p*-value for the comparison between survivor and non-survivor groups. Asterisks (*) indicate statistical significance (*p* < 0.05). CRP and IL-6 were measured at first presentation.
